# Evaluation of the suitability of six drought indices in naturally growing, transitional vegetation zones in Inner Mongolia (China)

**DOI:** 10.1371/journal.pone.0233525

**Published:** 2020-05-29

**Authors:** Yuqing Wang, Chengfu Zhang, Fan-Rui Meng, Charles P.-A. Bourque, Cunhou Zhang

**Affiliations:** 1 College of Desert Science and Engineering, Inner Mongolia Agricultural University, Hohhot, Inner Mongolia, P.R. China; 2 Faculty of Forestry and Environmental Management, University of New Brunswick, Fredericton, New Brunswick, Canada; 3 The Ecology and Agriculture Centre of Inner Mongolia, Hohhot, Inner Mongolia, P.R. China; Kerala University of Fisheries and Ocean Studies, INDIA

## Abstract

Naturally growing vegetation often suffers from the effects of drought. There exists a vast number of drought indices (DI’s) to assess the impact of drought on the growth of crops and naturally occurring vegetation. However, assessing the fitness of these indices for large areas with variable vegetation cover is often problematic because of the absence of adequate spatial information. In this study, we compared six DI’s to NDVI (the normalized difference vegetation index), a common indicator of vegetation occurrence and health based on satellite-acquired reflectance data. The study area covers an aridity gradient from forests to deserts along a 2,400-km-long section across the Inner Mongolia Autonomous Region of China. On an annual timescale, standardized precipitation index (SPI) was the most appropriate in assessing drought in steppes and deserts. On a seasonal timescale, the self-calibrated Palmer drought severity index (scPDSI) displayed the greatest sensitivity during the summer, but not during the other seasons. On a monthly timescale, scPDSI demonstrated the greatest sensitivity to the various vegetation zones (i.e., forests, steppes, and deserts) in June and July. Further analysis indicated that summer drought had a lag-effect on vegetation growth, which varied from one to six months according to the specific vegetation cover. The mixed response of DI’s to NDVI and the lag-effect in transitional vegetation on annual, seasonal, and monthly timescales were ascribed to differences in DI definition and the dominant plant species within the transitional cover. The current study has the potential to inform the drafting of selection criteria of DI’s for the study of drought-related impact on naturally growing vegetation at timescales from month to year.

## Introduction

Drought can lead to serious reduction in crop production, possibly affecting the socio-economic sustainability of communities [1]. Drought can lead to plant growth reductions by reducing the stomatal conductance and plant photosynthesis over the short term and plant biomass accumulation over the longer term [2]. Globally, drought causes billions of dollars of loss and adversely affects millions of people each year. Historically, China has been one of the most drought-prone countries of the world, with numerous records of long-lasting droughts that were responsible for widespread famines [3]. Reliable drought identification and prediction is hence vital for natural resources management in semiarid to arid parts of the world [4].

Drought indices (DI’s) have been extensively used in drought assessment, monitoring, and forecasting. Over the years, more than 150 DI’s have been developed [5]. Drobyshev et al. [6] studied the correlation of the standardized precipitation index (SPI), monthly drought code (MDC), Palmer drought severity index (PDSI), self-calibrated Palmer drought severity index (scPDSI) with direct monthly rainfall and fire frequency, and concluded that scPDSI was the most adequate to evaluate fire frequencies in the southern region of Sweden. Wang et al. [7] studied the pairwise relationships between SPI, SPEI, Palmer’s Z-index, PDSI, and soil moisture and found that: (i) with increased soil depth, soil moisture was more strongly correlated with DI’s at longer timescales; (ii) SPEI worked just as well as or better than SPI for all soil layers; (iii) the Z-index worked better than PDSI for shallow soils, with the opposite for deeper soils; and (iv) multi-component DI’s worked better than DI’s based on a two-layer bucket model. Tian et al. [8] evaluated six DI’s (i.e., PDSI, Palmer’s Z-index, precipitation percent normal, precipitation percentiles, SPI, and SPEI) in monitoring agricultural drought in southcentral USA and found no single index was able to capture all aspects of drought in the region.

Liu et al. [9] analyzed agricultural drought in the northern China plains using SPI, SPEI and PDSI, and found multiple indices were required to generate robust inferences of drought. Bai et al. [10] evaluated the applicability of scPDSI and SPEI using long-term satellite-acquired estimates of rainfall and the China Monthly Precipitation Analysis Product and found that SPEI was consistent with observations in eastern China, whereas scPDSI was found to provide inferior assessments of drought. Using monthly rainfall data at 6-, 12-, and 24-month timescales, Mahmoudi et al. [11] compared SPI, Percent of Normal Index (PNI), Z-Score Index (ZSI), Deciles Index (DI), CZI index (CZI), Effective Drought Index (EDI), and Modified China-Z Index (MCZI) and found through their research that SPI and EDI were best suited to monitor drought in Iran. Javed et al. [12] investigated drought evolution and spatiotemporal variations from 1982–2017 in crop-, forest-, grass-, and desertland in China using two remote sensing-based indices, i.e., (i) NDVI anomaly, and (ii) the vegetation condition index (VCI), and an evaluation of SPI using inputs from moderate resolution imaging spectroradiometer (MODIS) and long-term data records (LTDR). It revealed that a positive correlation existed between the DI’s (i.e., NDVI anomaly, VCI, and SPI) and rainfall for the different vegetation zones; with VCI performing better than NDVI anomaly. Differences in performance among DI’s resulted mainly because of differences in the physical environment (e.g., local to regional climatology, soils, and vegetation cover) that the indices were applied to [8] and the formulation of the indices. Determining which DI to use for particular regions and for particular purposes is not entirely clear. Given the range of DI’s currently available, it is essential to evaluate many of these indices to identify which ones are suitable for particular settings and purposes.

Ecologists are greatly concerned with the response and adaptability of terrestrial ecosystems to drought, particularly in water-stressed environments [13,14]. Drought affects both the function and structure of ecosystems [15]. Assessment of DI’s over large areas is often problematic because of the absence of adequate spatial information [16,17]. Currently, all research pertaining to drought impact on ecosystems has focused on agricultural ecosystems. Our study expands this research by assessing the response of six DI’s applied to natural ecosystems, from forests to steppes, to deserts, in northeastern China.

Remote sensing technologies provide an important source of spatiotemporal data in the study of vegetation dynamics and climate change, particularly because it offers near-continuous images over time and their convenience [18–20]. Vegetation response to drought over large, diverse areas is routinely observed with remote sensing techniques [21]. Healthy vegetation reflects more near infrared (NIR) and less red radiation. Normalized difference vegetation index (NDVI) is a normalized ratio of red to NIR spectral reflectance, i.e., (NIR-red)/(NIR+red). Prior research has shown that vegetation productivity, photosynthetic effective radiation, and vegetation coverage are strongly correlated to NDVI [22,23]. In practice, NDVI is often used in assessments of crop yields during periods of water-supply shortages and drought [24–26]. Nicholson et al. [27] used AVHRR-based estimates of NDVI and found particularly strong plant response to rainfall in the eastern desert steppes of Africa. Suzuki et al. [28] found a strong relationship between the spatial distribution of NDVI and seasonal change and climate factors in Inner Siberia. Barbosa et al. [22] analyzed NDVI changes in northeastern Brazil and found that values of NDVI in the area were greatly influenced by drought. Meng et al. [29] studied the characteristics of climate variability and NDVI from 1982–2000 in China, and observed strong correlation between NDVI and spatiotemporal variation in several key hydroclimatological variables. The primary objective of the research is to assess the suitability of different DI’s for different temporal resolutions, ranging from month to season, to year across a 2,400-km-long aridity gradient from semi-humid (forests) to arid climates (deserts) in northeastern to northcentral China.

## Methods and materials

### Study area

**Fig 1:**
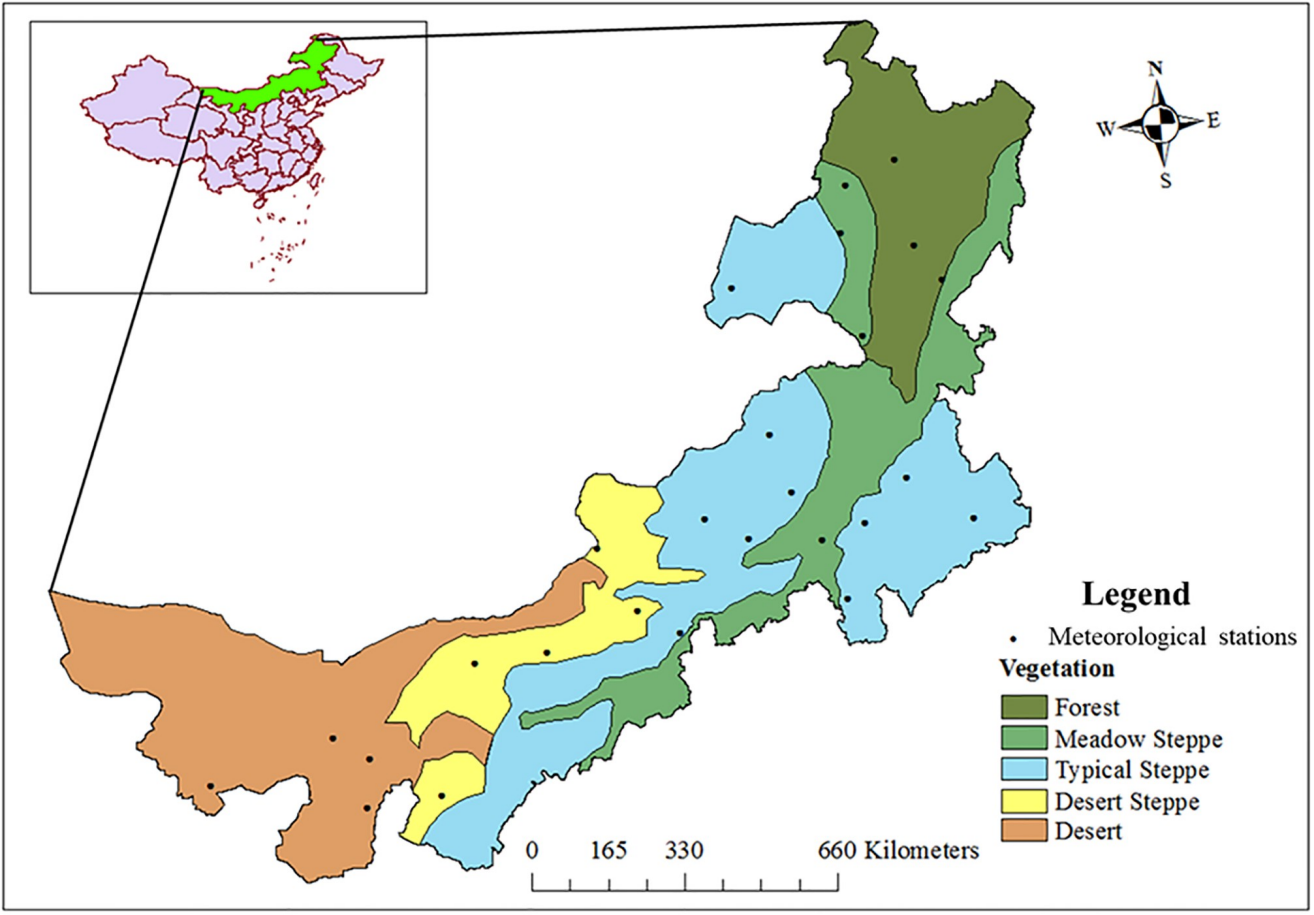
Vegetation zones and weather station locations across the aridity gradient from east to west. Reprinted from [32] under a CC BY license, with permission from the Chinese Journal of Plant Ecology, original copyright [1990].

The study area is located in Inner Mongolia Autonomous Region of China (37°24'-53°23'N, 97°12'-126°04'E), measuring 2,400 km from east to west and 1,700 km from north to south (Fig 1). The climate transitions from semi-humid to arid conditions can be seen from east to west of the study area. Vegetation zones along the gradient show clear transitions from forests, meadow steppes, typical steppes, desert steppes, and deserts, with annual temperature and cumulated precipitation increasing and decreasing from east to west, respectively (Fig 1). Plant species associations for each vegetation zone appear in Table 1. With differences in rooting depths, different plant species respond differently to drought. Owing to their deep roots, trees and some shrubs are largely resistant to long-lasting drought, compared with their shallow-rooted counterparts (e.g., grasses) [30].

**Table 1:** Annual temperature, precipitation, and dominant plant species within the five vegetation zones.

Ecoregion	Temperature (°C)			Precipitation (mm)	Dominant Species	Major Soil Types	Parent Material
	Mean	Maximum	Minimum				
Forest	-3.2	28.6	-31.2	450~500	Forest (*Larix gmelinii*, *Larix sibirica*, + *Betula platyphylla Suk*)	Black soil + Dark brown forest soil	Granite + Basalt
Meadow Steppe	-2.2	28.5	-31.6	350~500	Grass (*Leymus chinensis* + *Stipa baicalensis*)	Chernozem + Chestnut soil	Granite + Basalt
Typical Steppe	3	31.2	-24.5	300~400	Grass (*Stipa grandis* + *Stipa sareptana var*. *krylovii*)	Chestnut soil	Granite, Basalt, Gneiss, + Conglomerate
Desert Steppe	5.1	32.1	-23	135~311	Grass+shrub (*Stipa tianschanica var*. *klemenzii*, *Stipa breviflora*, *Stipa glareosa* + *sparsely distributed Caragana sinica*)	Chestnut soil + Brown calcic soil	Granite, Gneiss, Diorite, + Limestone
Desert	7.2	33.6	-19.2	45~215	Shrub (*Caragana sinica* + *Nitraria sphaerocarpa*)	Gray desert soil + Grey-brown desert soil	Granite, Diorite, Gneiss, Gabbro, + Sandstone

Intercontinental climate is characterized by cold and long winters, and dramatic swings in temperature during the spring and autumn. Inner Mongolia is one of the most sensitive regions to global climate change, experiencing substantial changes in climate over the past 50 years [31]. Frequency and severity of drought is expected to increase under continued global warming.

### Data source

Daily and monthly hydrometeorological data (2000–2015) from 26 weather stations in Inner Mongolia (Fig 1) were downloaded from the China Meteorological Data Network (http://data.cma.cn/, last accessed Nov. 14, 2018). These data and their derivatives served as input in the calculation of DI’s (see below). Vegetation evolution and dynamics was examined in terms of MODIS-based estimates of NDVI, downloaded from the Geospatial Data Cloud (http://www.gscloud.cn/, last accessed Nov. 14, 2018).

### Drought indices

The six DI’s considered here consist of SPI (standardized precipitation index), SPEI (standardized precipitation evapotranspiration index), MI (relative moisture index), Pa (precipitation anomaly percentage), K (Sielianinow coefficient), and scPDSI (self-calibrated Palmer drought severity index) (Table 2). The China Meteorological Administration currently uses some of these DI’s in the classification of meteorological drought throughout the country [31]. The K-index forms the basis for the Climate Regionalization in China and is widely used in assessments of climate change and associated drought, as well as assessments of rates of desertification across China [20,32]. Traits of the six DI’s include:

**SPI** is used to quantify precipitation deficits as anomaly percentile on multiple timescales. SPI depends on commonly available precipitation data and is relatively easy to implement in the assessment of drought severity at different timescales [33]. It is computed by fitting a Gamma probability density function to the frequency distribution of precipitation and transforming the Gamma distribution into a standardized normal distribution [34] (Table 2). The drought severity is defined by the probability of anomaly occurrence. Lloyd et al. [34] found SPI suitable for quantifying meteorological, hydrological, and agricultural drought.
**SPEI** is derived from SPI by standardizing the difference between water supply (precipitation) and water demand estimated from potential evapotranspiration [35, 36] (Table 2). The potential evapotranspiration is calculated with the Thornthwaite method, which takes into account air temperature and an annual heat index, based on monthly air temperature. The calculation of the index involves a climatic water balance implemented at different timescales, and adjusted to a log-logistic probability distribution. Because SPEI is based on an assessment of the water balance, it can be directly compared to scPDSI [35].
**MI** is calculated by dividing the difference between rainfall and potential evapotranspiration by the potential evapotranspiration during the same period [37] (Table 2); MI has been used to assess the degree of drought across multiple timescales.
**Pa** is calculated from the occurrence of drought caused by precipitation anomalies within a given period, which is calculated by dividing the difference between observed precipitation and normal (30-year mean) precipitation by the normal precipitation during the same period (Table 2). The index can distinguish dry events at monthly and annual timescales [38]. The index is simple to calculate and, as a result, is widely used.
**K** is calculated based on air temperature and rainfall amount during the growing season, whenever air temperature is ≥ 10°C [39]. The original equation coefficient of 0.10 was adjusted to 0.16 in adapting the method (Table 2) to China’s conditions [31].
**Palmer drought severity index (PDSI)** [40] is based on a sum of the current soil moisture anomaly and a fraction of the previous index value. The soil moisture anomaly is calculated based on an equation of water supply and demand and, as a result, it accounts for variables of precipitation, runoff, soil water storage, and evaporation [41]. The model requires data for air temperature, precipitation, and soil water content [42]. The resultant values are classed into 11 drought severities. With long-term calibration deficiencies and limited utility to areas of calibration, PDSI was further revised, giving rise to the self-calibrated PDSI (scPDSI; Table 2) [43]. The drought index calculated with scPDSI is generally more spatially compatible and accounts for extreme wet and dry events by taking into account frequencies of rare events.

**Table 2:** Drought indices considered in this study.

Drought indices	Equations	Variables
SPI (Standardized precipitation index) [33]	$\text{SPI}=\text{S(t}-\frac{{\text{c}}_{\text{0}}\text{+}{\text{c}}_{\text{1}}\text{t+}{\text{c}}_{\text{2}}{\text{t}}^{\text{2}}}{\text{1+}{\text{d}}_{\text{1}}\text{t+}{\text{d}}_{\text{2}}{\text{t}}^{\text{2}}\text{+}{\text{d}}_{\text{3}}{\text{t}}^{\text{3}}}\text{)}$ S = -1, if H(x) ≤ 0.5; S = 1 and H(x) = 1-H(x), if H(x)>0.5; H(x) = q+(1-q)G(x); q = P (x = 0) > 0.	H(x) is the cumulative probability of distribution;
		G(x) is the gamma distribution; G(x) is undefined for x = 0;
		P (x = 0) is the probability of zero precipitation, and x is precipitation in mm.
SPEI (Standardized precipitation evapotranspiration index) [5]	D_*i*_ = P_i_-PET_i_ D_i_ values are aggregated at different timescales, following the procedure for SPI. The difference $D_{i,j}^{k}$ in a given month *j* and year *i* depends on the timescale *k* chosen; D_i_-data series are subsequently fitted to a log-logistic probability distribution function.	P_i_ and PET_i_ are the precipitation and potential evapotranspiration for month *i* (both in mm);
		calculations of PET_i_ are based on the Thornthwaite method [46].
MI (Relative moisture index) [37]	$\text{MI}=\frac{\text{P-PET}}{\text{PET}}$	P is the rainfall in a given period (mm);
		PET is potential evapotranspiration (mm) during the same period.
Pa (Precipitation anomaly percentage) [38]	$\text{Pa}=\frac{\text{P-}\hat{\text{P}}}{\hat{\text{P}}}\;\times 100\%$	P is a period of precipitation;
		$\hat{\text{P}}$ is the average rainfall over the same period over successive years.
K (Sielianinow coefficient) [39]	$\text{K}\;=\;0.16\times \frac{\text{T}}{\text{P}}$	T and P are accumulated temperature (°C) and precipitation (mm), when temperature is ≥ 10oC.
scPDSI (Self-calibrated Palmer drought severity index) [40]	${\text{scPDSI}}_{\text{i}}=\text{0.755}{\text{scPDSI}}_{\text{i-1}}\text{+}\frac{\text{1}}{\text{1.63}}{\text{Z}}_{\text{i}}$ Z = Kd $\text{d}=\text{P-}\hat{\text{P}}$	Z is the Palmer moisture anomaly index;
		d is the moisture anomaly;
		P is the total monthly precipitation;
		$\hat{\text{P}}$ is a ‘climatologically appropriate’ precipitation amount for existing climatic conditions’;
		K is a climate coefficient (weight), determined by month and geographic location.

### Data analysis

Monthly and seasonal values of NDVI in each vegetation zone were averaged from the original 16-day and monthly data, respectively. Annual NDVI was, in turn, derived by averaging the growing-season data from May to August of each year. Values of NDVI were extracted within a 50-km radius circle around each weather station.

Monthly DI’s were calculated directly from equations referenced in Table 2. To investigate lag-effects (or hysteresis), pairwise-correlations between the six DI’s over two- to seven-month delays and NDVI of the current month were assessed.

Seasonal DI’s were calculated according to the four seasons, namely spring (March-May), summer (June-August), autumn (September-November), and winter (December-February of the following year). Considering precipitation in winter has the potential to impact vegetation growth in the following year, a year (or hydrological year) was defined here as the period from September to August of the following year. Annual and seasonal indices of K, Pa, SPI, and SPEI were calculated directly from corresponding total precipitation, mean air temperature, and total potential evapotranspiration, whereas annual and seasonal indices of MI and scPDSI were calculated by averaging their monthly values.

Indices of SPI, SPEI, and scPDSI were calculated with a python-script developed for the National Integrated Drought Information System of the USA (www.drought.gov/drought/climate-and-drought-indices-python, last accessed Nov. 14, 2018). Potential evapotranspiration in MI (Table 2) was calculated based on the Penman-Monteith equation, using software developed for the Food and Agriculture Organization of the United Nations (http://www.fao.org/land-water/databases-and-software/eto-calculator/en/, last accessed Nov. 14, 2018). Indices of K, Pa, and MI were calculated in Excel (*v*. 2016, Microsoft Corporation). All subsequent correlations were determined in SPSS *v*. 22 (International Business Machines Corporation).

## Results

### Annual pairwise-correlations

Table 3 provides the annual pairwise-correlations between the six DI’s and NDVI for the five vegetation zones. Pairwise-correlations between the six DI’s and NDVI, from highest to lowest, were associated with typical steppes, followed by meadow steppes, desert steppes, deserts, and forests. In forests, pairwise-correlations between all DI’s and NDVI were generally low (r < 0.46) and statistically not significant (*p*-value > 0.05). For the three steppes, pairwise-correlations were generally high, i.e., maximum r = 0.90, 0.92, and 0.84, respectively, with *p*-values < 0.01. In desert zones, pairwise-correlations were generally greater than those for forests and lower for steppes (maximum r = 0.68, *p*-value < 0.01). From averaged pairwise-correlations for the three steppes, the greatest pairwise-correlation was 0.85 for SPI, followed by 0.84 for SPEI, and 0.83 for Pa.

**Table 3:** Annual correlation coefficients of NDVI and DI within the five vegetation zones.

Vegetation	SPI	SPEI	MI	Pa	K	scPDSI	Maximum
Forests	0.45	0.43	0.32	0.45	0.46	0.33	0.46
Meadow Steppes	0.90**	0.84**	0.23	0.81**	0.72**	0.47	0.90
Typical Steppes	0.82**	0.92**	0.60*	0.85**	0.69**	0.57*	0.92
Desert Steppes	0.84**	0.77**	0.75**	0.83**	0.70**	0.69**	0.84
Deserts	0.68**	0.46	0.62*	0.63**	0.38	0.54*	0.68
Average for Steppes	0.85	0.84	0.53	0.83	0.70	0.58	
Average for All Vegetation Zones	0.74	0.68	0.50	0.71	0.59	0.52	

* Signifies correlation is significant at the 0.05 level,

** at the 0.01 level.

### Seasonal pairwise-correlations

Seasonal pairwise-correlations between the six DI’s and NDVI for the five vegetation zones are given in Table 4. Generally, NDVI had low correlation with all DI’s during the spring and autumn, compared to their corresponding values in summer. In some cases, correlations were negative in spring and autumn.

**Table 4:** Seasonal correlation coefficients between NDVI and DI within the five vegetation zones. The “Maximum” in column 8 is given as an absolute value.

Vegetation	Season	SPI	SPEI	MI	Pa	scPDSI	Maximum
Forests	Spring	-0.38	-0.15	-0.06	-0.06	-0.34	0.38
	Summer	0.29	0.28	0.11	0.28	0.31	0.31
	Autumn	-0.09	-0.11	-0.43	-0.02	0.43	0.43
Meadow Steppes	Spring	0.17	-0.09	-0.11	0.26	0.05	0.26
	Summer	0.68**	0.68**	0.59*	0.67**	0.65**	0.68
	Autumn	0.21	0.07	-0.24	0.04	0.26	0.26
Typical Steppes	Spring	0.44	0.21	0.31	0.31	0.47	0.47
	Summer	0.73**	0.81**	0.81**	0.73**	0.88**	0.88
	Autumn	0.41	0.43	0.44	0.41	0.54*	0.54
Desert Steppes	Spring	0.01	-0.16	-0.07	0.06	0.21	0.21
	Summer	0.61*	0.69**	0.61*	0.63**	0.82**	0.82
	Autumn	0.38	0.21	0.32	0.41	0.36	0.38
Deserts	Spring	0.37	0.31	0.05	0.13	0.11	0.37
	Summer	0.14	0.55*	0.46	0.43	0.71**	0.71
	Autumn	-0.09	0.21	-0.14	-0.13	-0.07	0.21
Average for Steppes	Summer	0.67	0.73	0.67	0.68	0.78	
Average for All Vegetation Zones	Summer	0.49	0.60	0.52	0.55	0.67	

* Signifies correlation is significant at the 0.05 level,

** at the 0.01 level.

In forests, pairwise-correlations were generally low (i.e., r < 0.4; Table 4) and statistically not significant. In meadow steppes, SPI and SPEI had the greatest correlation in summer, i.e., r = 0.68. In typical and desert steppes, scPDSI had the greatest correlation with NDVI, yielding 0.88 and 0.82, respectively, followed by SPEI in summer, with r = 0.81 and 0.69. In deserts, scPDSI had the greatest correlation with NDVI in summer (r = 0.71).

### Monthly pairwise-correlations

**Fig 2:**
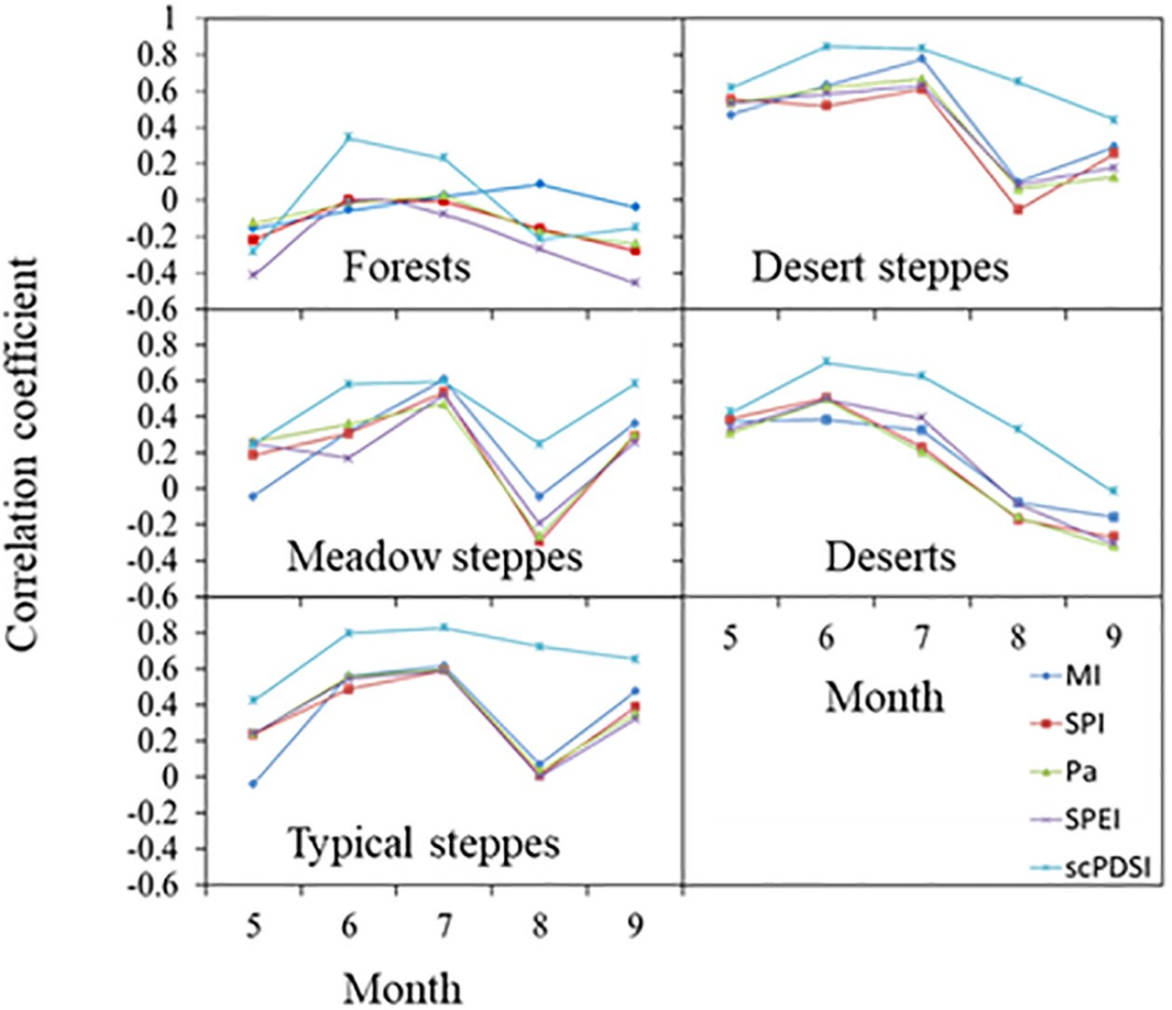
Pairwise-correlations of monthly drought indices of SPI, SPEI, MI, Pa, and scPDSI as a function of NDVI across the five vegetation zones.

Fig 2 provides pairwise-correlations of current-month DI’s with current-month NDVI. Across months, irrespective of vegetation zone, correlations were least in May. Similar to the annual timescale, DI’s were the least correlated with NDVI for forests during the growing season. For meadow steppes, correlation was lowest in August, compared to September. A similar trend was found for typical and desert steppes when based on SPI, SPEI, MI, and Pa. Only scPDSI produced correlations greater in August than in September. In deserts, correlations based on all DI’s were greatest in June, and subsequently decreased from July to September. Among DI’s and irrespective of vegetation zone, scPDSI had the strongest correlation with NDVI in June and July (Fig 2).

### Lag-effect of drought

**Fig 3:**
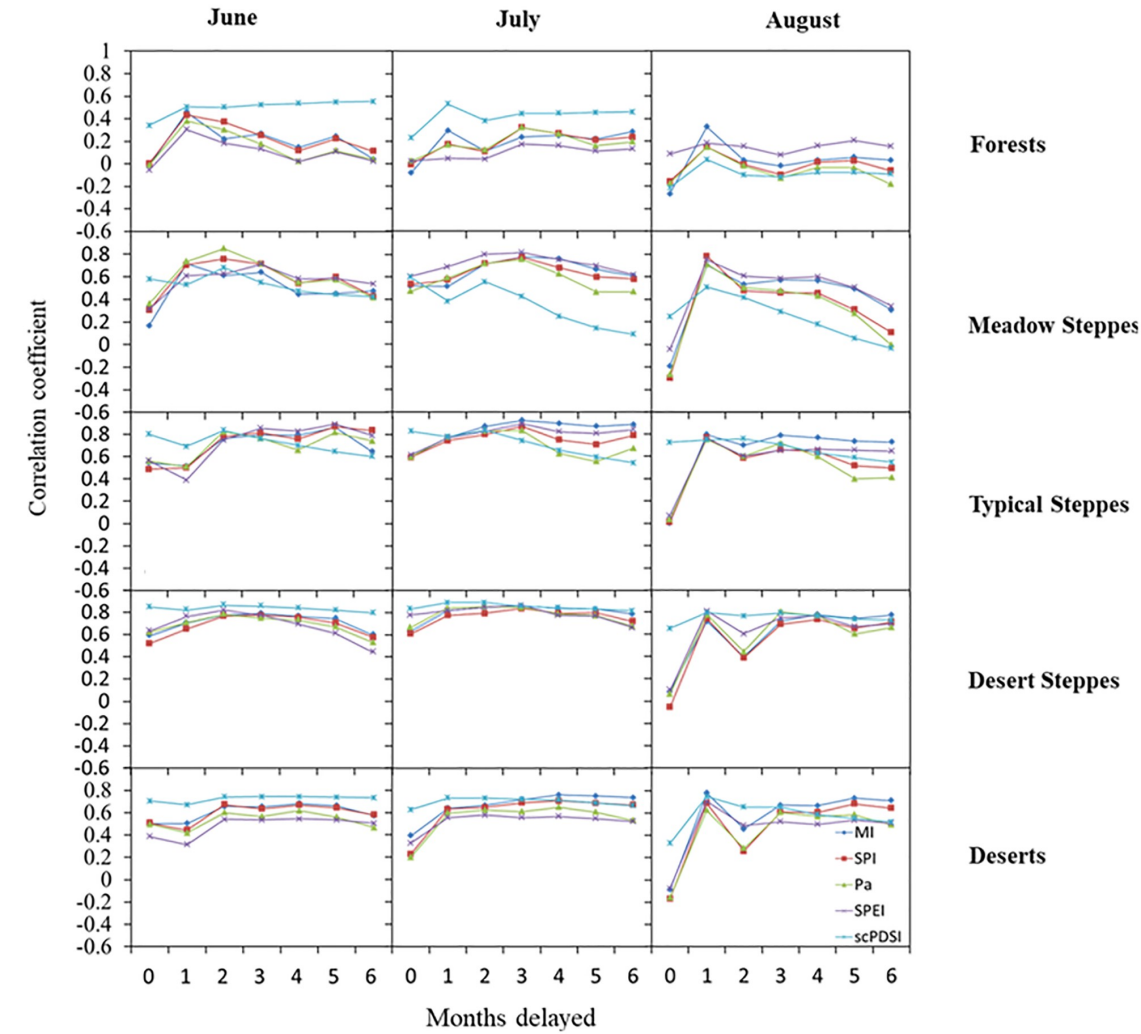
Pairwise-correlations of lagged monthly drought indices with corresponding NDVI during the months of June, July, and August across the five vegetation zones.

Pairwise-correlations between NDVI and lagged DI’s were much greater than between NDVI and DI’s during the current-month for all vegetation zones (Fig 3). Lag times among DI’s varied.

In forests, lagged scPDSI had the greatest correlation in June and July among the six DI’s, with maximum correlation occurring after about six months of lag in June and about one month of lag in July. For the other DI’s, however, maximum correlation was found to occur after about one month of lag in June and August. In meadow steppes, lagged SPI and Pa had high correlations with NDVI, after about two months of lag in June and about three months in July. In August, however, maximum correlation existed between NDVI and all DI’s lagged by one month. In typical steppes, greatest pairwise-correlations with SPI, SPEI, MI, and Pa and NDVI occurred when the DI’s were lagged by about five months in June, three months in July, and one month in August. Generally, all correlations were greatest for meadow and typical steppes. In desert steppes and deserts, lagged scPDSI had the greatest correlation with NDVI, when compared to the other DI’s. For all vegetation zones, NDVI in August were more strongly correlated with the DI’s when lagged by one month, than when not lagged (Fig 3).

## Discussion

### Occurrence of drought over the study period

Located in the interior of Eurasia, Inner Mongolia is affected by continental climate and frequently affected by drought. During the study period from 2000–2015, drought affected many areas of the study region differently. Over the study period, six out of the 16 years (i.e., 2002, 2003, 2012, 2013, 2014, and 2015) were found unaffected by drought [44]. The dataset amassed for this study provides a strong basis for the current examination of drought and suitability of the various DI’s.

### Implications of timescale

On an annual timescale, SPI and SPEI were shown to have a much stronger correlation with NDVI in grasslands (steppes). This is consistent with results from a study that assessed the impact of drought on wheat yield with the same two DI’s [5]. If annual drought is the primary focus, SPI is the preferable DI for steppes and deserts.

Pairwise-correlations between DI’s and NDVI were highest in summer and lowest in spring and fall in all vegetation zones, as a result of low temperatures prevailing during the latter two seasons, i.e., low temperatures, not available soil water, limit vegetation growth during these seasons [8,45]. Comparing different DI’s, scPDSI had stronger correlations with NDVI in the summer for most vegetation zones, except the meadow steppes. In these steppes, SPI and SPEI had stronger correlation with NDVI in summer (r = 0.68), about equivalent to the correlation between scPDSI and NDVI (r = 0.65). Based on these results, scPDSI is a superior DI for summer conditions. This conclusion applies equally well to monthly data, where correlation between scPDSI and summer NDVI were higher than any other pairwise-comparisons across all vegetation zones, with the exception of forests (August only, Fig 2). Current-month scPDSI has added advantages of being a good measure of monthly drought and being based solely on current-month information, offers a more generalized indicator of drought.

### Lag-effect of drought

The lag-effect suggests that the effects of drought on vegetation potentially become more important as time elapses. This coincides with observations that the response of NDVI lags changes in temperature and precipitation [46–49]. This lag-effect occurs not only as a function of leaf accumulation, but also because of the amount of water stored in the soil complex. Leaf biomass, especially in grasses including in annual and perennial plant species, is an accumulated value and determined by past drought conditions. Rainfall and meltwater infiltration into the deeper soil is generally available to vegetation over extended periods, causing plant growth, and therefore NDVI, to lag the incidence of infiltration into the soil.

Lag times vary as a function of vegetation zone. Pairwise-correlations between lagged DI’s (by 2–7 months) and NDVI were consistently highest for desert steppes and deserts and lowest for forests, meadows, and typical steppes. Grasses in steppes are adapted to short-term drought, whereas shrubs with their various adaptations to dry air and soil conditions are much more resistant to long-term drought [50]. This plant association to drought is consistent with our belief that grasses in steppes are more sensitive to seasonal and monthly drought, and shrubs in deserts are more sensitive to annual drought.

Capacity to store water in soils may help explain the observed lag-effect of drought. Drought indices of SPI, SPEI, Pa, and MI account for only the water supply and demand components of the water budget (i.e., precipitation and evapotranspiration), resulting in the indices being skillful at only accounting for present-time drought response. This may explain why SPI, SPEI, Pa, and MI provide improved correlations with NDVI when lagged. This introduced lagging of the indices effectively compensates for the absence of soil water storage and potential carry-over effects in their explanation of drought (Table 2). Unlike the two-component indices, scPDSI incorporates soil water storage in its definition of drought (Table 2). Due to its three-component formulation, scPDSI is regarded as the better measure of monthly drought across vegetation zones. This conclusion is consistent with findings from prior studies on the utility of DI’s; for instance, the study by Chang et al. [51]. This finding has significant implication for gauging the timing, development, and persistence of drought.

### Vegetation response to drought

In forests, all DI’s had weaker correlations with NDVI across all timescales, even after taking into account the lag-effect of drought. Forests, located near mountain ranges, receive additional water from snowmelt, improving overall growing conditions for forests downslope [50]. The deep roots of trees can access water from deep in the ground and large quantities of carbohydrates and nutrients stored in the roots can cause forests to be less vulnerable to severe, prolonged meteorological drought [32]. In forests, water is normally not a limiting factor.

The steppes are located in the semiarid to arid parts of the aridity gradient. With their shallow rooting systems, grasses are fundamentally more sensitive to reductions in precipitation [52]. Grasses in steppes absorb water from the upper soil layers, and as a result rely on frequent, short pulses of meteoric water input [53]. In steppes, the six DI’s were effective at capturing drought across the three timescales and month-long lag times considered. The lag-effect of drought at the monthly timescale indicated that longer timescales were necessary to impact vegetation growth in the steppes. All told, lagged SPI, SPEI, MI, and Pa provided reasonably good measures of monthly drought. In considering lag on NDVI response, Pa, SPEI and SPI lagged by one to three months, provided the strongest measure of drought in meadow and typical steppes, whereas current-month scPDSI provided a more reliable measure of ongoing drought in desert steppes.

In deserts, shrubs are the dominant vegetation (Table 1). Desert plants generally have lower transpiration rates, with enormous capacity to absorb water from the ground [53]. Shrubs in deserts adapt to dry conditions with their unique features to limit water losses by, for example: (i) curling their leaves or growing hairs on their surfaces; (ii) storing water in their fleshy or succulent parts; and (iii) accessing water with their vast network of horizontal and vertical roots [54]. Many shrubs in deserts utilize water from the deeper soil and rely on moisture that is replenished with each successive rain pulse [50].

Besides the impact of abiotic variables, growth of natural vegetation can be adversely affected by non-abiotic factors, such as human, livestock, and insect activity. During the 13 years from 2001–2013, NDVI predicted with temperatures and precipitation data from Inner Mongolia was significantly correlated with MODIS-based estimates of NDVI, which suggest that human, grazing, and other biological effects on plant growth were less significant than the effects associated with prevailing hydrometeorological factors [55]. In grassland and desert regions of Inner Mongolia, livestock is the primary factor affecting grass development and growth. Based on results from the Century model [56], growth of grass was shown to be inversely proportional to the severity of grazing.

## Conclusions

Until now, most of the studies of this kind have focused on drought effects in agricultural crops; few studies have examined these effects on naturally growing, transitional vegetation. In this study, we analyzed the pairwise-correlations between six common DI’s and NDVI, as a measure of vegetation occurrence and response. This study found that:

At an annual timescale, there were strong pairwise-correlations between the six DI’s and NDVI for semiarid steppes, weak but statistically significant correlations for deserts, and no correlation for forests. Compared with the different DI’s, SPI is the most appropriate to assess drought in steppes and deserts, followed by SPEI and Pa.

At a seasonal timescale, there were strong pairwise-correlations between the six DI’s and NDVI during the summer, but not during the spring or autumn. The scPDSI-index was most fitting for typical steppes, desert steppes, and deserts during the summer, followed by SPEI; SPI and SPEI were most appropriate for meadow steppes. None of the six DI’s considered were appropriate for forests.

At a monthly timescale, there were significantly stronger correlations between NDVI and scPDSI of the current month, compared with the other DI’s in June and July. During the summer, NDVI had stronger correlation with DI’s of previous months (accounting for a lag-effect of drought) than their corresponding current-month values. The monthly lag-effect is obvious for all DI’s, except scPDSI. The lag ranged from one to six months and was distinct for the different vegetation zones, varying from month-to-month. The lag-effect was most obvious in August, irrespective of vegetation zone.

Based on our analysis, SPI could be viewed as the most appropriate to monitor drought on an annual basis and scPDSI, for summer drought in semiarid steppes and deserts. We recommend that users of these indices pay attention to the lag-effect of drought and the functional response of local vegetation at a monthly timescale. With climate change, drought has the potential to affect naturally growing vegetation more severely than in the past. With the 16-year dataset provided here, it is fairly difficult to conclude about the overall suitability of the DI’s over longer time horizons.

## References

[1] Piao S, Ciais P, Huang Y, Shen Z, Peng S, Li J, et al The impacts of climate change on water resources and agriculture in china. Nature 2010; 467(7311):43–51. 20811450 10.1038/nature09364

[2] Yuan W, Luo Y, Li X, Liu S, Yu G, Zhou T, et al Redefinition and global estimation of basal ecosystem respiration rate. Global Biogeochemical Cycles 2011; 25(4):1441–1458.

[3] Zou X, Zhai P, Zhang Q. Variations in droughts over china: 1951–2003. Geophysical Research Letters 2005; 32(4):353–368.

[4] Shamshirband S. Clustering project management for drought regions determination: a case study in Serbia. Agricultural and Forest Meteorology 2015; 200(200):57–64.

[5] Vicente-Serrano SM, Beguería S, López-Moreno JI. A multiscalar drought index sensitive to global warming: the standardized precipitation evapotranspiration index, Journal of Climate 2010; 23(7):1696–1718.

[6] Drobyshev I, Niklasson M, Linderholm HW. Forest fire activity in Sweden: climatic controls and geographical patterns in 20th century. Agricultural and Forest Meteorology 2012; 15:174–186.

[7] Wang H, Rogers JC, Munroe DK. Commonly used drought indices as indicators of soil moisture in China, Journal of Hydrometeorology 2015; 16(3):1397–1408.

[8] Tian L, Yuan S, Quiring SM. Evaluation of six indices for monitoring agricultural drought in the south-central United States. Agricultural and Forest Meteorology 2018; 249:107–119.

[9] Liu X, Zhu X, Pan Y, Bai J, Li S. Performance of different drought indices for agriculture drought in the North China Plain. Journal of Arid Land 2018; 10(4):207–516.

[10] Bai X, Wen S, Wu X, Wang P. Applicability of long-term satellite-based precipitation products for drought indices considering global warming. Journal of Environmental Management 2020; 255:109846. 31747628 10.1016/j.jenvman.2019.109846

[11] Mahmoudi P, Rigi A, Kamak MM. A comparative study of precipitation-based drought indices with the aim of selecting the best index for drought monitoring in Iran. Theoretical and Applied Climatology 2019; 10.1007/s00704-019-02778-z.

[12] Javed T, Yao N, Chen X, Suon S, Li S. Drought evolution indicated by meteorological and remote-sensing drought indices under different land cover types in China. Environmental Science and Pollution Research 2019; 10.1007/s11356-019-06629-2. 31828700

[13] Liu D, Yu C, Zhao F. Response of the water use efficiency of natural vegetation to drought in northeast china. Journal of Geographical Sciences 2018; 28(5): 611–628.

[14] Kang W, Wang T, Liu S. The response of vegetation phenology and productivity to drought in semi-arid regions of northern china. Remote Sensing 2018; 10(5):727(1–15).

[15] Wu X, Liu H, Li X, Ciais P, Babst F, Guo W, et al Differentiating drought legacy effects on vegetation growth over the temperate Northern Hemisphere. Glob Chang Biology 2017; 24(1):1–13. 10.1111/gcb.13920 28973825

[16] Heim RR. A review of twentieth-century drought indices used in the united states, Bulletin of the American Meteorological Society 2002; 83(8):1149–1165.

[17] Mishra AK, Singh VP. A review of drought concepts. Journal of Hydrology 2010; 391(1): 202–216.

[18] Potter CS, Brooks V. Global analysis of empirical relations between annual climate and seasonality of NDVI. International Journal of Remote Sensing 1998; 19(15):2921–2948.

[19] Parmesan C, Yohe G. A globally coherent fingerprint of climate change impacts across natural systems. Nature 2003; 421(6918):37–42. 12511946 10.1038/nature01286

[20] Piao S, Mohammat A, Fang J, Cai Q, Feng J. NDVI-based increase in growth of temperate grasslands and its responses to climate changes in China. Global Environmental Change 2006; 16(4):340–348.

[21] Dong C, MacDonald GM, Willis K, Gillespie TW, Okin GS, Williams AP. Vegetation responses to 2012–2016 drought in northern and southern California. Geophysical Research Letters 2019; 10.1029/2019GL082137.

[22] Barbosa HA, Huete AR, Baethgen WE. A 20-year study of NDVI variability over the Northeast Region of Brazil, Journal of Arid Environments 2006; 67(2):288–307.

[23] Ma W, He J, Yang Y, Wang X, Liang C, Anwar M, et al Environmental factors covary with plant diversity–productivity relationships among Chinese grassland sites. Global Ecology and Biogeography 2010; 19(2):233–243.

[24] Bausch WC. Remote sensing of crop coefficients for improving the irrigation scheduling of corn, Agricultural Water Management 1995; 27(1):55–68.

[25] Haroon MA, Zhang J, Yao F. Drought monitoring and performance evaluation of MODIS-based drought severity index (DSI) over Pakistan. Natural Hazards 2016; 84(2):1349–1366.

[26] Zhao A, Zhang A, Cao S, Liu X, Liu J, Cheng D. Responses of vegetation productivity to multi-scale drought in loess plateau, china. Catena 2018;163: 165–171.

[27] Nicholson SE, Davenport ML, Malo AR. A comparison of the vegetation response to rainfall in the Sahel and East Africa, using normalized difference Vegetation index from NOAA AVHRR, Climatic Change 1990; 17(2–3):209–241.

[28] Suzuki R, Nomaki T, Yasunari T. Spatial distribution and its seasonality of satellite-derived Vegetation index (NDVI) and climate in Siberia. International Journal of Climatology 2001; 21(11):1321–1335.

[29] Meng M, Ni J, Zong M. Impacts of changes in climate variability on regional Vegetation in China: NDVI-based analysis from 1982 to 2000. Ecological Research 2011; 26(2): 421–428.

[30] Meng M, Ni J, Zhang Z. Aridity index and its applications in geo-ecological study. Acta Phytoecologica Sinica 2004; 28(6):853–861.

[31] Zhang C, Liu H, Song Y, Liao Y, Duan J, Cai W, et al Classification of Meteorological Drought (GB/T 20481–2017). China Meteorological Press, Beijing (in Chinese). 2017.

[32] Committee of Physical Regionalization of the Chinese Academy of Sciences. Synthetic physical regionalization of China. 1959; Science Press, Beijing. (In Chinese).

[33] McKee TB, Doesken, NJ, Kleist J. The relationship of drought frequency and duration to timescales. 1993; In: Proceedings of the 8th Conference in Applied Climatology. American Meteorological Society Boston, MA, USA pp.179–184.

[34] Lloyd-Hughes B, Saunders MA. A drought climatology for Europe. International Journal of Climatology 2002; 22(13):1571–1592.

[35] Vicente-Serrano SM, Beguería S, Lorenzo-Lacruz J, Camarero JJ, López-Moreno JI, Azorin-Molina C, et al Performance of drought indices for ecological, agricultural, and hydrological applications. Earth Interact 2012; 16 (10):1–27.

[36] Thornthwaite CW, Mather JR. The water balance. Publications in Climatology. Lab. Drexel Institute of Technology 1955; 8(1):1–104.

[37] Yao YB, Qiang Z, Wang JS, Shang JL, Wang Y, Shi J, et al Spatiotemporal change of spring drought in Southwest China, Journal of Animal and Plant Sciences 2015; 25(3):260–269.

[38] Yao N, Li Y, Lei T, Peng L., Drought evolution, severity and trends in mainland China over 1961–2013, Science of the Total Environment 2017; 73:616–617. 10.1016/j.scitotenv.2017.10.327 29107781

[39] Radzka E, Rymuza K, Lenartowicz T. Analysis of hydrothermal conditions and their impact on early potato yields. Journal of Ecological Engineering 2015; 16(2):120–124.

[40] Palmer W. “Meteorological Drought” Research Paper, US Department of Commerce Weather Bureau, Washington DC, No. 45, 1965.

[41] Benjamin L-H. A drought climatology for Europe, International Journal of Climatology 2002; 22(13):1571–1592.

[42] Zargar A, Sadiq R, Naser B, Khan FI. A review of drought indices. Environ. Rev. 2011; 19:333–349. 10.1139/a11-013.

[43] Wells N, Goddard S, Hayes MJ. A self-calibrating palmer drought severity index. Journal of Climate 2004; 17(12):2335–2351.

[44] Tong S. Spatio-temporal variations and prediction of meteorological drought in Inner Mongolia under climate change. 2019; PhD Thesis. Northeast Normal University, Changchun, P.R. China.

[45] Zhang C, Wang M, Wulanbater, Jiang X. Responses of ANPP to climate change in Inner Mongolia typical steppe—a simulation study. Acta Botanica. Boreali-Occidentalia Sinica 2012; 32(6):1229–1239 (in Chinese).

[46] Wang J, Price K, Rich P. Spatial patterns of NDVI in response to precipitation and temperature in the central Great Plains. International Journal of Remote Sensing 2001; 22(18):3827–3844.

[47] Wang J, Rich PM, Price KP. Temporal response of NDVI to precipitation and temperature in the Central Great Plains, USA. International Journal of Remote Sensing 2003; 24(11):2345–2364.

[48] Guo Q, Hu Z, Li S, Yu G, Sun X, Zhang L, et al Contrasting responses of gross primary productivity to precipitation events in a water- limited and a temperature-limited grassland ecosystem. Agricultural and Forest Meteorology 2015; 214-215(3):169–177.

[49] Arredondo T, Garcìa-Moya E, Huber-Sannwald E, Loescher HW, Delgado-Balbuena J, Luna-Luna M. Drought manipulation and its direct and legacy effects on productivity of a monodominant and mixed-species semi-arid grassland. Agricultural & Forest Meteorology 2016; 223:132–140.

[50] D'Odorico P, Porporato A. Ecohydrology of arid and semiarid ecosystems: an introduction. In: D'Odorico and Porporato (Eds.), Dryland Ecohydrology 2006; pp. 1–10.

[51] Chang J, Wang ZW, Li Y, Han GD. Relationship between aboveground net primary productivity and precipitation and air temperature of three plant communities in Inner Mongolia grassland. Journal of Inner Mongolia University 2010; 41(6):689–694 (In Chinese).

[52] Peng J, Dong W, Yuan W, Zhang Y. Responses of grassland and forest to temperature and precipitation changes in Northeast China. Advances in Atmospheric Sciences 2012; 29(5):1063–1077.

[53] Wang Q. Studies on water ecological adaptability of dominant desert plants on the eastern Alashan Western Erdos area. M.Sc. Inner Mongolia University, Hohhot, P.R. China. 2008.

[54] Pospíšilová J, Larcher W. Physiological Plant Ecology. Ecophysiology and stress physiology of functional groups. Fourth Edition. Biologia Plantarum 2003; 47(4):500–500.

[55] Li X. Variations and impact factors of vegetation cover Inner Mongolia based on the MODIS-NDVI. M.Sc. Inner Mongolia University. Hohhot. P.R. China. 2014.

[56] Parton WJ, McKeown B. Kirchner V, Ojima D. CENTURY users' manual, Natural Resource Ecology Laboratory, Colorado State University, Ft. Collins 1992.

